# HSP90 activity is required for MLKL oligomerisation and membrane translocation and the induction of necroptotic cell death

**DOI:** 10.1038/cddis.2015.386

**Published:** 2016-01-14

**Authors:** A V Jacobsen, K N Lowes, M C Tanzer, I S Lucet, J M Hildebrand, E J Petrie, M F van Delft, Z Liu, S A Conos, J-G Zhang, D C S Huang, J Silke, G Lessene, J M Murphy

**Affiliations:** 1 The Walter and Eliza Hall Institute of Medical Research, Parkville, VIC, Australia; 2 Department of Medical Biology, University of Melbourne, Parkville, VIC, Australia; 3 Department of Pharmacology and Therapeutics, University of Melbourne, Parkville, VIC, Australia

## Abstract

Necroptosis is a caspase-independent form of regulated cell death that has been implicated in the development of a range of inflammatory, autoimmune and neurodegenerative diseases. The pseudokinase, Mixed Lineage Kinase Domain-Like (MLKL), is the most terminal known obligatory effector in the necroptosis pathway, and is activated following phosphorylation by Receptor Interacting Protein Kinase-3 (RIPK3). Activated MLKL translocates to membranes, leading to membrane destabilisation and subsequent cell death. However, the molecular interactions governing the processes downstream of RIPK3 activation remain poorly defined. Using a phenotypic screen, we identified seven heat-shock protein 90 (HSP90) inhibitors that inhibited necroptosis in both wild-type fibroblasts and fibroblasts expressing an activated mutant of MLKL. We observed a modest reduction in MLKL protein levels in human and murine cells following HSP90 inhibition, which was only apparent after 15 h of treatment. The delayed reduction in MLKL protein abundance was unlikely to completely account for defective necroptosis, and, consistent with this, we also found inhibition of HSP90 blocked membrane translocation of activated MLKL. Together, these findings implicate HSP90 as a modulator of necroptosis at the level of MLKL, a function that complements HSP90's previously demonstrated modulation of the upstream necroptosis effector kinases, RIPK1 and RIPK3.

Necroptosis is an inflammatory, caspase-independent form of regulated cell death characterised by loss of cellular membrane integrity and release of cytoplasmic contents.^1^ It is believed to have evolved as a defence mechanism against viruses;^2, 3^ however, there is increasing evidence that deregulated necroptosis has a role in the pathogenesis of a range of inflammatory, autoimmune and neurodegenerative diseases.^4, 5, 6, 7, 8^ Reduced capacity to undergo necroptosis has been correlated to increased aggressiveness of cancers;^9, 10^ and therapeutic initiation of necroptosis is currently being investigated as a cancer therapy.^11, 12^ Additionally, there is emerging evidence that the necroptotic signalling pathway has a general role in the modulation of inflammation.^13, 14, 15, 16, 17^ As such, unravelling the molecular events governing necroptosis, and potential avenues for therapeutic intervention, is of enormous interest.

Necroptosis is initiated through activation of death receptors, such as Tumour Necrosis Factor Receptor 1 (TNFR1), or through microbial activation of pattern recognition receptors, such as Toll-like receptors or intracellular viral DNA sensors.^3, 18, 19, 20^ Receptor ligation initiates a signalling cascade, whereby Receptor Interacting Protein Kinase (RIPK)-3 oligomerises and is phosphorylated, a process known to be regulated by association with other effectors, such as the protein kinase RIPK1, TIR-domain-containing adapter-inducing IFN-*β* (TRIF), or DNA-dependent activator of IFN regulatory factors (DAI), via their RIP Homotypic Interaction Motifs (RHIMs).^2, 21, 22^ Once activated, RIPK3 phosphorylates the pseudokinase domain of Mixed Lineage Kinase domain-Like (MLKL), the most downstream known obligate effector of the necroptotic signalling pathway, to induce its activation.^23, 24^ MLKL phosphorylation is thought to trigger a molecular switch,^25, 26, 27^ leading to the unleashing of the N-terminal executioner four-helix bundle (4HB) domain,^28^ MLKL oligomerisation and translocation to cellular membranes where cell death occurs via an incompletely-understood mechanism.^28, 29, 30^

Molecular chaperones have an integral role in modulating both the structure and function of proteins. One such chaperone is heat-shock protein 90 (HSP90), which interacts with a diverse group of protein ‘clients', the largest group comprising the kinases and pseudokinases, with 50% of the human kinome estimated to interact with HSP90.^31^ These interactions are dependent on the recognition of the kinase or pseudokinase domain by the HSP90 co-chaperone Cdc37, which enables HSP90 to confer protein stabilisation, assist in late-stage folding and conformational modifications, and mediate intracellular transport.^32, 33, 34, 35^

It has already been demonstrated that the necroptotic pathway is subject to modulation by HSP90. RIPK1 is well established as an HSP90 client protein, with a number of studies finding HSP90 inhibition affects both the stability and function of RIPK1 and promotes an apoptotic phenotype.^36, 37, 38, 39, 40, 41^ More recently, RIPK3 was also identified as an HSP90 client.^2, 42, 43^ Surprisingly, HSP90 inhibition did not markedly impact RIPK3 abundance or stability, but rather was essential for RIPK3's necroptotic functions, such as phosphorylation of MLKL.^42^ However, whether MLKL itself is a client of HSP90 has not been investigated.

In this study, using a phenotypic screen for small-molecule inhibitors of MLKL-driven cell death, we identified HSP90 as a modulator of necroptosis that functions on, or downstream of, the terminal effector, MLKL. HSP90 inhibition did not markedly reduce levels of MLKL in human U937 or mouse dermal fibroblasts, suggesting instead that HSP90 has an active role in governing MLKL-mediated cell death. This idea is supported by our finding that cell death driven by the S345D activated mutant of MLKL in *Ripk3*-deficient fibroblasts in the absence of necroptotic stimuli was suppressed by three distinct chemical classes of HSP90 inhibitor, but MLKL abundance was not impacted by HSP90 inhibition. Although our data indicate that MLKL binds HSP90 weakly or transiently, HSP90 activity was essential for the assembly of MLKL into high molecular weight complexes and the membrane translocation known to precede cell death. These findings suggest an expanded role for HSP90 in regulating necroptosis, and further our understanding of the mechanisms controlling MLKL-mediated cell death.

## Results

### HSP90 modulates necroptosis independent of MLKL activation

**Figure 1:**
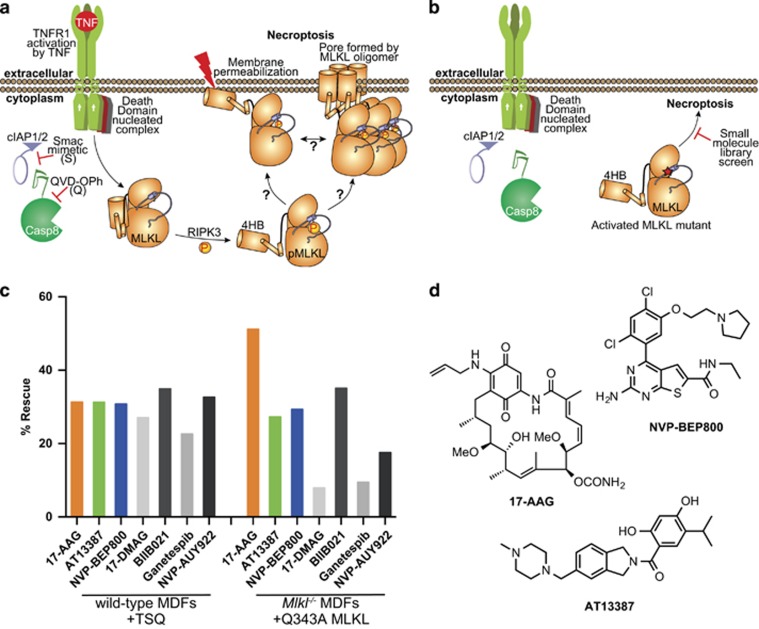
Hsp90 inhibitors can block necroptosis downstream of MLKL activation. (**a**) A schematic of the necroptosis pathway. TNF (T) stimulates the TNFR1; cIAP1/2 activity is blocked with Smac mimetic (S); and the pan-caspase inhibitor, QVD-OPh (Q), blocks caspase-8 activity. This leads to RIPK3 activation and subsequent phosphorylation and activation of MLKL. (**b**) The activated MLKL mutant Q343A initiates cell death in the absence of TSQ stimulation or RIPK3 activation enabling a screen for inhibitors of necroptosis downstream of MLKL activation. (**c**) Cells were pretreated for 2 h with a library of 438 compounds with annotated mechanisms of action at 1 *μ*M, then necroptosis was stimulated in wild-type MDFs with TSQ and expression of Q343A MLKL was induced in *Mlkl*^−/−^ MDFs with doxycycline. Percentage rescue was determined using a CellTiter Glo assay, and normalised against TSQ/doxycycline stimulation and DMSO treatment. The data represent the HSP90 hits from each screen, and are the average of two technical replicates. (**d**) Chemical structures of the three compounds selected for further analysis

To identify small-molecule inhibitors of necroptosis, we performed phenotypic screens with a library comprising 438 small molecules with well-annotated mode of actions in both wild-type murine dermal fibroblasts (MDFs) treated with necroptotic stimuli (Figure 1a) and *Mlkl*^−/−^ MDFs inducibly expressing the Q343A activated mutant of MLKL, which undergoes cell death in the absence of stimuli (Figure 1b). Necroptosis was induced in wild-type MDFs using conventional necroptotic stimuli: tumour necrosis factor (TNF; T), to activate TNF receptor 1; the Smac mimetic, Compound A (S), to inhibit cIAP (cellular inhibitor of apoptosis proteins)-mediated ubiquitylation of RIPK1 and permit its participation in cell death signalling; and the pan-caspase inhibitor, Q-VD-OPh (Q), to inhibit caspase-8 and permit necroptosis (Figure 1a). In parallel, the same library of 438 compounds was tested in *Mlkl*^−/−^ MDFs reconstituted with the Q343A mutant MLKL, which we previously showed to induce cell death in the absence of necroptotic stimuli, independently of RIPK3 activity (Figure 1b).^25^ Compounds that conferred protection against necroptotic cell death were identified by measuring cell viability using a CellTiter Glo assay.

All seven HSP90 inhibitors within our small-molecule library protected wild-type MDFs from undergoing TSQ-induced necroptosis and, strikingly, provided some protection to *Mlkl*^−/−^ MDFs undergoing stimulus-independent death driven by the Q343A MLKL mutant (Figure 1c). Approximately 30% rescue from TSQ-driven cell death in wild-type MDFs was conferred by each of the seven HSP90 inhibitors (Figure 1c). Four of the seven inhibitors, 17-AAG, AT13387, NVP-BEP800 and BIIB021, also provided substantial protection from Q343A MLKL-mediated cell death. These four HSP90 inhibitors belong to distinct chemical classes, ruling out any off-target effects of these small molecules on the necroptotic pathway. These results, therefore, implicate HSP90 as a modulator of the necroptosis pathway downstream of MLKL activation.

We selected the most potent inhibitor, the geldanamycin analogue 17-AAG, along with two other inhibitors from distinct chemical classes; AT13387 and NVP-BEP800 (Figure 1d), to further explore the role of HSP90 in necroptosis. We selected compounds from three distinct chemical classes to eliminate the possibility that their inhibition of necroptosis occurs via off-target effects. Because these compounds all inhibit HSP90 through engagement of the ATP binding site,^44, 45, 46^ we performed a thermal shift assay to assess whether the anti-necroptotic function of HSP90 inhibitors might arise from off-target binding to the MLKL ATP binding pocket. However, none of these three compounds induced a marked thermal shift of either the mouse or human MLKL pseudokinase domains, or full-length mouse or human MLKL. This suggests that HSP90 inhibitors do not act directly on MLKL (Supplementary Figure 1), but rather function via inhibition of HSP90 activity.

### HSP90 inhibition subtly reduced endogenous MLKL protein levels

**Figure 2:**
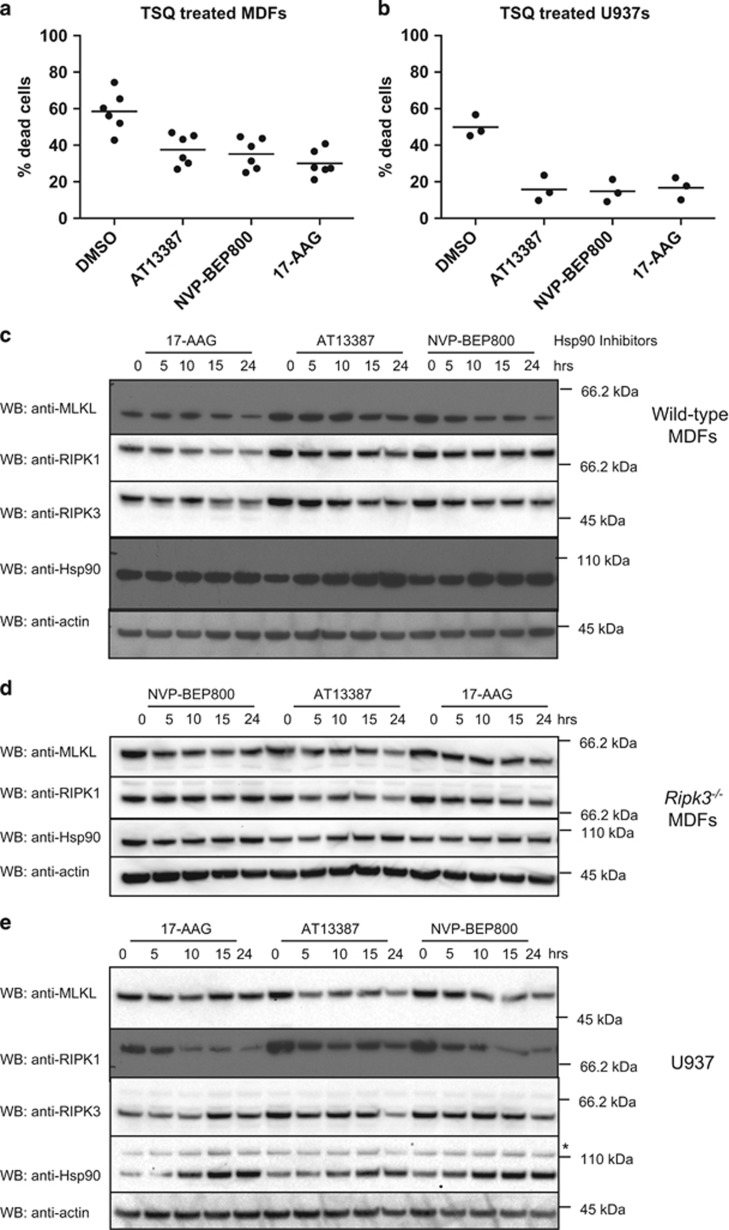
MLKL levels are modestly reduced by Hsp90 inhibition. (**a**) MDFs were pretreated for 1 h with AT13387 (1 *μ*M), NVP-BEP800 (125 nM), 17-AAG (250 nM) or DMSO, then necroptosis was induced with TSQ. After 24 h, propidium iodide (PI) uptake was measured using flow cytometry. Each data point represents results from one of three independent cell lines tested in two experiments and solid bar indicates average (*n*=6). (**b**) U937 cells were pretreated for 1 h with AT13387 (2 *μ*M), NVP-BEP800 (1 *μ*M), 17-AAG (500 nM) or DMSO, then necroptosis was induced with TSQ. After 24 h, PI uptake was measured using flow cytometry. Each data point represents results from the U937 cell line tested in three independent experiments and solid bar indicates average (*n*=3). (**c** and **d**) Wild-type (**c**) or *Ripk3*^−/−^ (**d**) MDFs were treated with AT13387 (1 *μ*M), NVP-BEP800 (125 nM) or 17-AAG (250 nM) for the indicated times, then cell lysates analysed with western blotting using the indicated antibodies. Data are representative of three independent experiments. (**e**) U937 cells were treated with AT13387 (2 *μ*M), NVP-BEP800 (1 *μ*M) or 17-AAG (500 nM) over a 24-h time course, then cell lysates analysed with western blotting using the indicated antibodies. Data are representative of three independent experiments. *Represents non-specific band at ~110 kDa

We performed titrations of 17-AAG, AT13387 and NVP-BEP800 on wild-type MDFs and U937, a human histiocytic lymphoma cell line, to determine the optimal concentrations for further analysis (Supplementary Figure 2). Cells were pretreated for 1 h with each inhibitor before induction of necroptosis by addition of TSQ, and cell death quantified by propidium iodide uptake after 24 h by flow cytometry. All three HSP90 inhibitors provided approximately 20–30% protection from TSQ-mediated killing in MDFs, which was consistent with the results of our primary phenotypic screen (Figure 2a). In U937, pre-treatment with inhibitors was less intrinsically toxic at higher concentrations of inhibitors than in MDFs and resulted in near complete protection (Figure 2b).

A common function of HSP90 is to protect client proteins from degradation. Upon loss of HSP90 protection, client proteins revert to a partially folded state, making them more likely to aggregate and undergo proteasomal degradation.^39, 47^ As such, a decrease in protein level in response to HSP90 inhibition is suggestive of HSP90 client status. We examined the effects of HSP90 inhibition in wild-type and *Ripk3*^−^^/^^−^ MDFs and found there was indeed a decrease in MLKL protein levels, which was subtle but became most apparent after 15–24 h of treatment (Figures 2c and d). This was accompanied by a decrease in protein levels of other known clients in the necroptosis pathway, RIPK1 and RIPK3. RIPK1 levels were also noticeably reduced in U937 cells following HSP90 inhibition (Figure 2e). These results suggest that MLKL stability is not dramatically affected by HSP90 inhibition. We therefore sought other explanations for loss of MLKL killing activity.

### HSP90 is required for MLKL protein function

**Figure 3:**
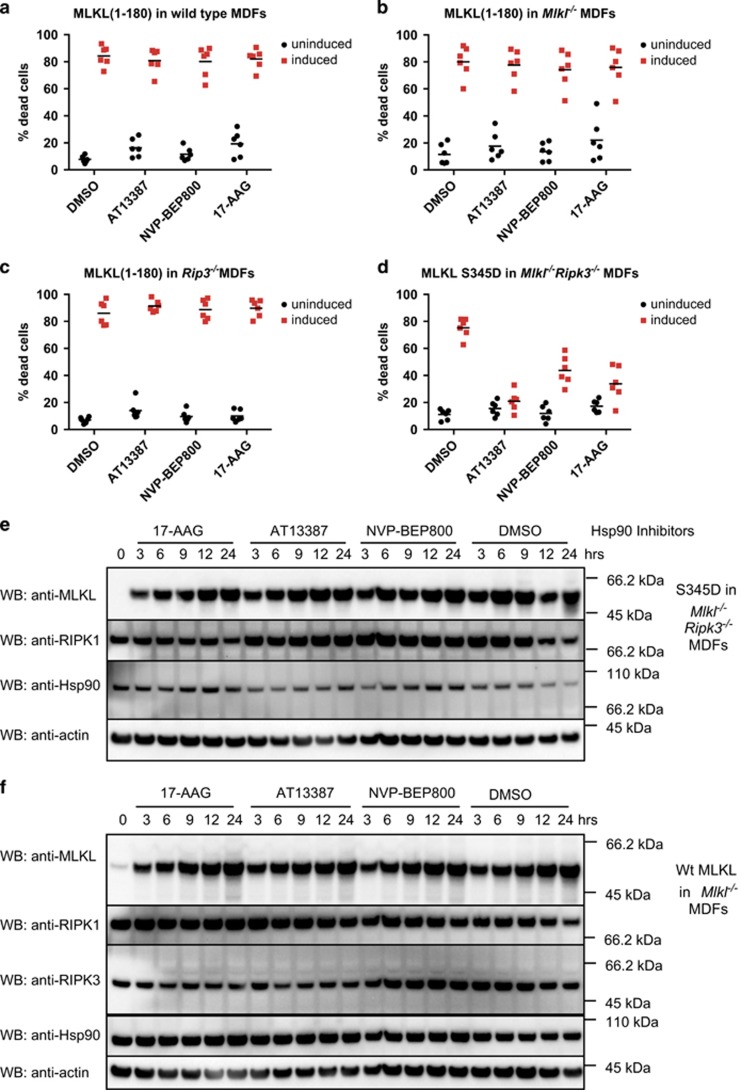
Hsp90 inhibition affects MLKL activity. (**a**–**c**) Wild-type (**a**), *Mlkl*^−/−^ (**b**) or *Ripk3*^−/−^ (**c**) MDFs were pretreated for 1 h with AT13387 (1 *μ*M), NVP-BEP800 (125 nM), 17-AAG (250 nM) or DMSO, then expression of MLKL(1–180) was induced using 50 ng/ml doxycycline. After 24 h, PI uptake was measured using flow cytometry. Two experiments were performed using three independent cell lines (*n*=6). (**d**) *Mlkl*^−/−^ *Ripk3*^−/−^ MDFs were pretreated with HSP90 inhibitors as described above, then expression of MLKL S345D was induced using 50 ng/ml doxycycline. After 24 h, PI uptake was measured using flow cytometry. Two experiments were performed using three independent cell lines (*n*=6). (**e** and **f**) Cells were pretreated for 1 h with AT13387 (1 *μ*M), NVP-BEP800 (125 nM), 17-AAG (250 nM) or DMSO, then expression of MLKL S345D in *Mlkl*^−/−^*Ripk3*^−/−^ double knockout MDFs (**e**) or endogenous MLKL in *Mlkl*^−/−^ MDFs (**f**) was induced using 50 ng/ml doxycycline. Treatment was performed over 24 h at the times shown, then cell lysates analysed with western blotting using the indicated antibodies. Data are representative of three independent experiments

We have proposed that the auto-activating MLKL Q343A mutant is in an activated conformation that exposes the N-terminal executioner four-helix bundle (4HB) domain thereby leading to cell death.^25^ Consistent with this proposal, expression of the 4HB domain of MLKL is sufficient to kill cells.^28^ We therefore hypothesised that HSP90 might directly regulate the killing activity of 4HB domain. To test this, we inducibly expressed a truncated MLKL construct encoding residues 1–180, which incorporates the executioner 4HB domain, in wild-type MDFs pretreated with our panel of HSP90 inhibitors. However, HSP90 inhibition did not prevent 4HB domain-induced death (Figure 3a). The amount of cell death was also unaffected when the MLKL(1–180) construct was expressed in either *Mlkl*^−/−^ or *Ripk3*^−/−^ MDFs (Figures 3b and c). These data suggest that HSP90 acts at the level of MLKL activation, probably via a mechanism that depends on the presence of the C-terminal pseudokinase domain of MLKL.

To explore this hypothesis, we used a previously-described MLKL S345D mutant that mimics RIPK3-mediated activation loop phosphorylation and thereby causes stimulus-independent activation of MLKL.^25^ To completely eliminate any possible contribution of RIPK3, this protein was inducibly expressed in *Ripk3*^−/−^*Mlkl*^−/−^ double knockout (DKO) MDFs. In contrast to the cell death induced by the 4HB domain alone, but consistent with our original screen, all three inhibitors showed substantial protection from S345D MLKL-mediated killing (Figure 3d).

Although this result suggested that HSP90 was involved in the conversion of MLKL into its activated form downstream of RIPK3, mutated proteins have been shown to have a greater reliance on HSP90 for stabilisation.^48, 49^ To evaluate whether reduced stability of the S345D MLKL mutant following HSP90 inhibition might explain the striking reduction in necroptosis, we evaluated levels of this mutant over a 24-h time period. Interestingly, S345D levels were not notably reduced by any HSP90 inhibitor (Figure 3e). We also evaluated the effect of HSP90 inhibition on the levels of inducibly-expressed wild-type MLKL in *Mlkl*^−/−^ MDFs, and found protein levels to be comparable to those observed in *Mlkl*^−/−^ MDFs expressing the S345D MLKL mutant (Figure 3f). Together, these data indicate that HSP90 has a role in converting MLKL into its activated form, rather than in regulating its abundance.

### HSP90 activity is necessary for MLKL oligomerisation and translocation to the membrane

**Figure 4:**
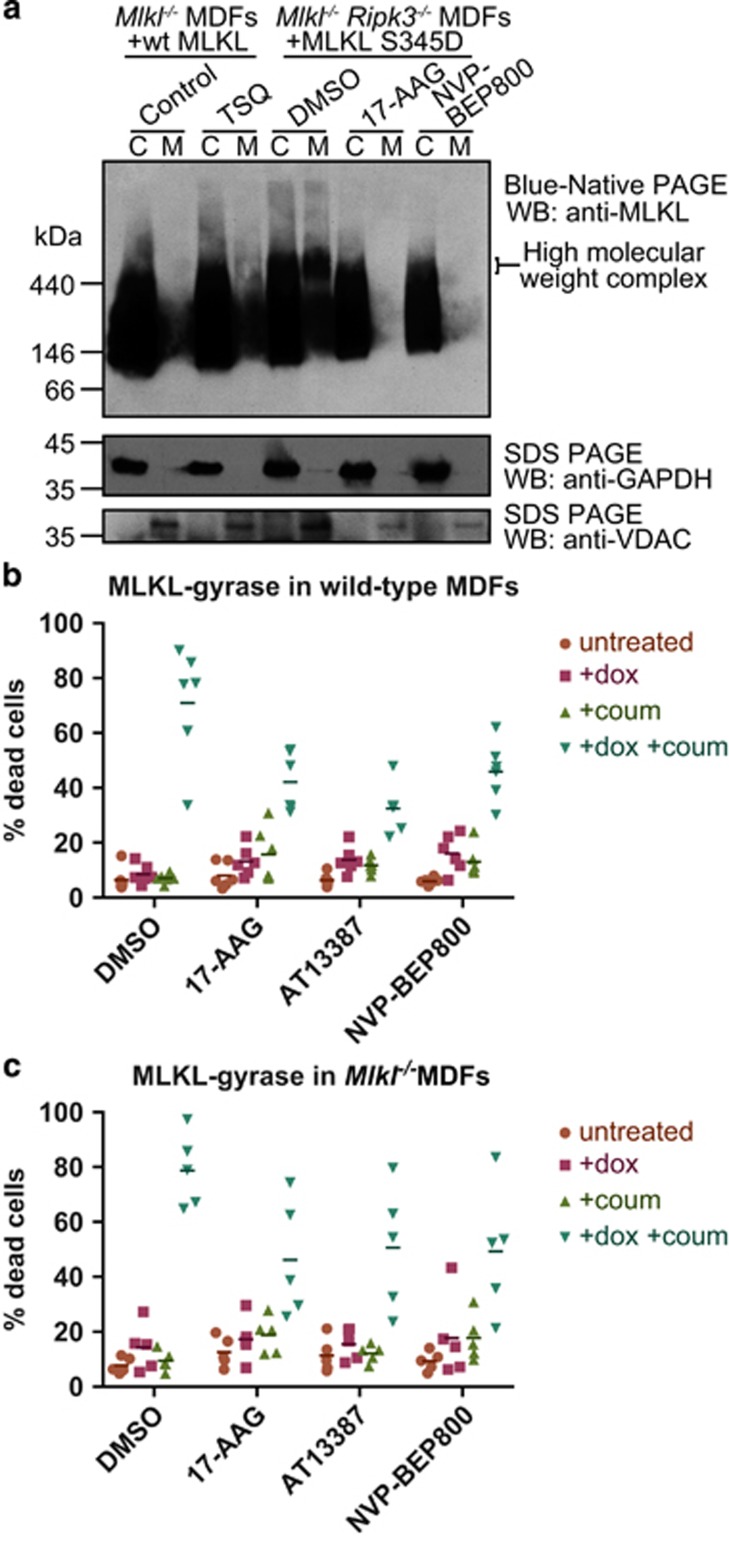
HSP90 is required for oligomerisation and membrane translocation of MLKL. (**a**) *Mlkl*^−/−^ *Ripk3*^−/−^ MDFs were pretreated for 1 h with NVP-BEP800 (125 nM), 17-AAG (250 nM) or DMSO then expression of MLKL S345D was induced using 50 ng/ml doxycycline. Cells were harvested when ~40% of DMSO-treated cells were dead (~7 h after induction). Expression of wild-type MLKL in *Mlkl*^−/−^ MDFs was induced using 50 ng/ml doxycycline for 3 h, then cells were either left untreated (Control) or treated with TSQ to induce necroptosis. Cells were harvested when cell death was ~40% in TSQ-treated cells (~5 h after TSQ treatment). Cell lysates were then separated into cytoplasmic (C) or membrane (M) fractions, and separated using Blue Native PAGE. Levels of MLKL were analysed using western blotting. Data are representative of three independent experiments using two biologically independent cell lines. (**b** and **c**) Expression of wild-type full-length MLKL fused via its C-terminus to DNA gyraseB was expressed in wild-type (**b**) or *Mlkl*^−/−^ (**c**) MDFs using 50 ng/ml doxycycline (+dox) and were dimerised via treatment with 700 nM coumermycin (+coum). Cells were pretreated for 1 h with AT13387 (1 *μ*M), NVP-BEP800 (125 nM), 17-AAG (250 nM) or DMSO, before MLKL-gyrase expression was induced with dox. After 24 h, propidium iodide (PI) uptake was measured using flow cytometry. Each data point represents results from one of three independent cell lines tested in two experiments, and solid bar indicates mean (*n*=6 for wild-type MDFs; *n*=5 for *Mlkl*^−/−^ MDFs)

A critical step in MLKL-induced killing entails membrane translocation, and HSP90 has been shown to regulate trafficking of proteins to the plasma membrane.^32, 50^ We therefore asked whether HSP90 might have an important role in mediating MLKL translocation to the membrane fraction. To investigate this possibility, we treated *Ripk3*^−/−^*Mlkl*^−/−^ double knockout MDFs expressing S345D MLKL with either DMSO or HSP90 inhibitors for 7 h and performed Blue Native PAGE on the cytoplasmic and membrane fractions (Figure 4a). Consistent with the HSP90 inhibitors' ability to protect from necroptosis, they greatly reduced the amount of S345D MLKL present in the membrane fraction and prevented the formation of the high molecular weight complexes that we have previously described as a hallmark of necroptotic cell death.^28, 51^ Because HSP90 has been established as a regulator of RIPK1,^36, 37, 38, 39, 40, 41^ RIPK3,^2, 42, 43^ and now MLKL (herein), it was unsurprising that treatment of TSQ-stimulated U937 cells with the HSP90 inhibitors, 17-AAG or NVP-BEP800, similarly inhibited the assembly of endogenous MLKL into high molecular weight species and membrane translocation, as assessed by Blue Native PAGE (Supplementary Figure 3a).

Although we found that the membrane translocation of the S345D activated MLKL mutant was blocked by HSP90 inhibitors, it remained of interest whether HSP90 may also contribute to the assembly of MLKL oligomers. To further examine this possibility, we used a construct in which full-length wild-type mouse MLKL was C-terminally fused to the *E. coli* DNA GyraseB domain, which can be dimerised by treatment with the divalent antibiotic, coumermycin.^52, 53^ As described elsewhere, doxycycline-induced expression of MLKL-gyrase in wild-type or *Mlkl*^−/−^ MDFs did not induce cell death unless MLKL-gyrase was dimerised by the addition of coumermycin (Figures 4b and c).^53^ The extent of cell death induced by forced dimerisation of MLKL-gyrase was impaired, but still measurable, in the presence of HSP90 inhibitors (Figures 4b and c). These data suggest that HSP90 may function in the assembly of higher order complexes, either by promoting an MLKL conformation that can assemble into oligomers or by recruitment of auxiliary proteins, following MLKL activation by RIPK3. It is possible, however, that HSP90 may also contribute to downstream necroptosis effects, such as mediating or promoting MLKL membrane translocation.

### MLKL interacts transiently with HSP90 via the co-chaperone, Cdc37

**Figure 5:**
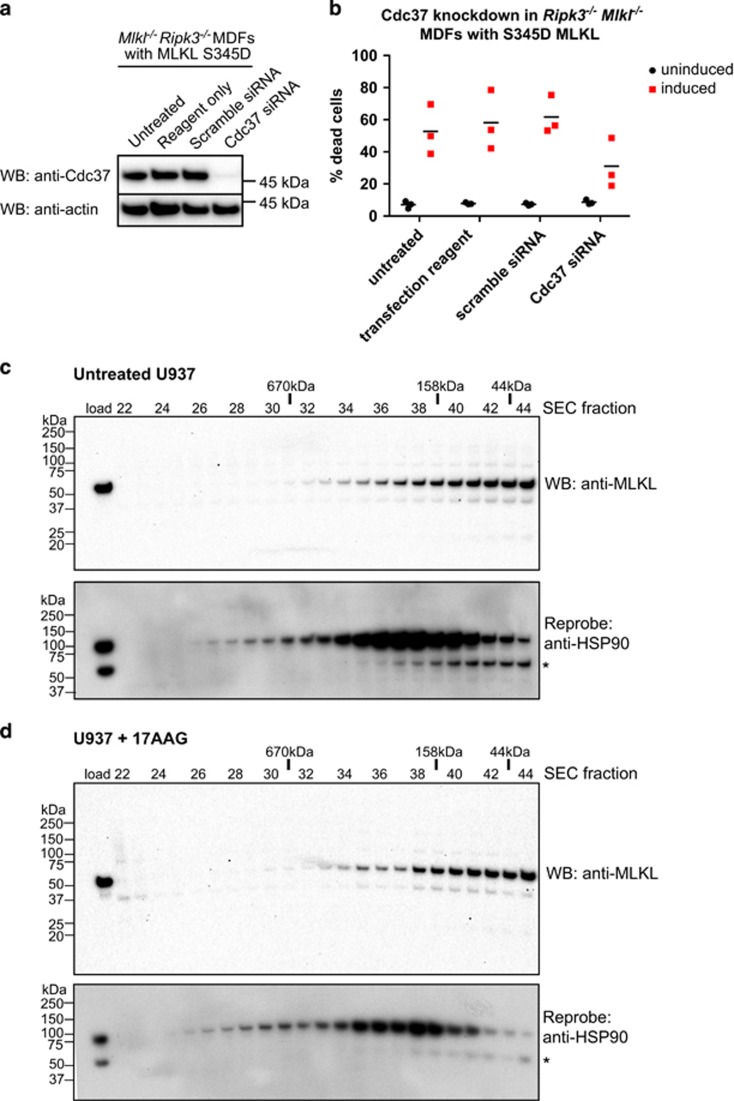
MLKL transiently interacts with HSP90 via the Cdc37 co-chaperone. (**a** and **b**) *Mlkl*^−/−^ *Ripk3*^−/−^ MDFs stably transduced with a lentiviral construct encoding S345D MLKL were untreated, treated with transfection reagent only, or transfected with scrambled or Cdc37 siRNA pools. Cdc37 knockdown was observed in Cdc37 siRNA-treated cells relative to untreated, transfection reagent and scrambled siRNA-treated controls by western blot (**a**). Only Cdc37 siRNA knockdown conferred protection from S345D MLKL-mediated death (**b**). (**c** and **d**) Lysates of U937 cells incubated with DMSO (**c**) or 17-AAG (500 nM, **d**) were resolved by Superose-6 10/300 size-exclusion chromatography (SEC). Fractions were subjected to SDS-PAGE and western blotted for MLKL (upper panels) before reprobing for HSP90 (lower; * corresponds to residual signal from MLKL blots). The SEC fraction number is shown above the blots along with the elution position of molecular weight standards

The finding that HSP90 activity on MLKL relied on the presence of the MLKL pseudokinase domain (Figures 3a–d) led us to test whether the kinase/pseudokinase co-chaperone, Cdc37, might serve as an adaptor for HSP90 recruitment. We used siRNA to knockdown Cdc37 expression in *Mlkl*^−/−^ *Ripk3*^−/−^ MDFs expressing the S345D activated MLKL mutant (Figure 5a), which led to a corresponding reduction in S345D MLKL-mediated cell death (Figure 5b).

Although these data indicate that Cdc37 serves as a co-chaperone to bridge HSP90 and MLKL, it remained of interest to better understand the nature of the interaction between MLKL and HSP90. To this end, using a S345D MLKL construct bearing a C-terminal StrepII tag, we attempted to co-immunoprecipitate HSP90 from MDF cell lysates. Unexpectedly, we did not observe a robust interaction between S345D mouse MLKL-StrepII and HSP90 in StrepTactin pulldown experiments (Supplementary Figure 3b), leading us to speculate that the MLKL/HSP90 interaction may be weak or transient. As a result, we sought to assess whether an interaction could be detected between endogenous MLKL and HSP90 under non-necroptotic conditions. We reasoned that interpreting whether MLKL binds HSP90 under necroptotic conditions was potentially confounded by earlier reports that MLKL is recruited to the necrosome,^23, 24^ a complex containing RIPK1 and RIPK3, which themselves are known HSP90 interactors. We further reasoned that owing to the decrease in endogenous protein observed in unstimulated cells treated with HSP90 inhibitors (Figure 2), MLKL may interact with HSP90 before induction of necroptosis, analogous to reported observations for RIPK1 and RIPK3.^42^ To address this, we subjected lysates from U937 cells treated with DMSO (Figure 5c) or 17-AAG (Figure 5d) to Superose-6 size-exclusion chromatography fractionation and used western blot analyses to determine whether HSP90 and MLKL co-elute. The elution profiles indicated that HSP90 and MLKL elute as separate, but overlapping, peaks. The almost identical profiles observed in control (DMSO; Figure 5c) and 17-AAG-treated (Figure 5d) lysate fractionations confirmed that HSP90 activity had no bearing on the elution profile of MLKL. Collectively, these data suggest that the effect of HSP90 on MLKL is mediated via a weak or transient, rather than stable, interaction.

## Discussion

Although active kinases are well established as HSP90 clients, the impact of this chaperone on pseudokinase stability and function is less well described. Several pseudokinases, including PSKH2, NPR2/ANP*β*, EphB6, KSR1, KSR2 and ErbB3/HER3, were implicated as HSP90 clients in a study of a large portion of the human kinome.^31^ However, the impact of HSP90 on pseudokinase function has only been described for a handful of these intriguing proteins. Ryk, EphB6 and integrin linked kinase (ILK) have been shown to be stabilised through interaction with HSP90,^33, 54, 55, 56^ while ErbB3/HER3 maturation and cell-surface translocation were found to rely on HSP90 activity.^57^ In this study, we show that necroptosis induction by the pseudokinase MLKL is regulated by HSP90 activity.

HSP90 frequently stabilises its client proteins and in its absence, or when it is inhibited, client proteins are more rapidly turned over by the ubiquitylation/proteasomal machinery.^58^ Although we observed a modest reduction in protein levels of endogenous MLKL in response to three different HSP90 inhibitors, this was not as dramatic as the reduction in levels of a well-known HSP90 client, RIPK1, in the same cells. Furthermore, when MLKL was ectopically expressed, HSP90 inhibition had no discernible effect on its levels. Based on our observations that HSP90 interaction with MLKL is likely to be weak or transitory, rather than stable, it would be surprising if HSP90 markedly contributed to the maintenance of MLKL protein levels. This transient interaction is reminiscent of that between HSP90 and another TNF signalling effector, the I*κ*B kinase holocomplex,^59^ which like the HSP90/MLKL interaction examined in the present work, similarly relied on the co-chaperone Cdc37.

It is widely accepted that MLKL must oligomerise and translocate to membranes in order to kill cells.^28, 29, 30, 60, 61^ In mouse cells, RIPK3 phosphorylates S345 in the ‘activation loop' of MLKL's pseudokinase domain and we have proposed that this phosphorylation event causes a conformational change that leads to exposure of the N-terminal 4HB domain, which allows it to translocate to, and permeabilise, membranes.^25, 28^ However, the mechanism for MLKL translocation and membrane permeabilisation is currently unclear. Consistent with a model in which RIPK3-mediated phosphorylation of MLKL S345 induces exposure of the executioner 4HB domain, expression of the phosphomimetic S345D MLKL mutant induces MLKL incorporation into high molecular weight membrane complexes, membrane localisation and stimulus-independent cell death,^51^ while expression of the 4HB domain alone likewise promotes necroptosis.^28^ HSP90 inhibitors prevented S345D MLKL-induced necroptosis, but did not prevent 4HB domain-induced death. These results suggest that HSP90 function is required before MLKL-mediated membrane permeabilisation: in promoting a conformation change necessary for MLKL activation, assembly of MLKL into high molecular weight complexes and/or translocation to membranes. Because HSP90 has been shown to contribute to dimerisation and activation of conventional protein kinases, such as LIM Kinase 1,^62^ we examined whether HSP90 inhibitors blocked the cell death arising from forced dimerisation of MLKL. Cell death induced by MLKL forced dimerisation was partially blocked by HSP90 inhibitors, indicating that HSP90 serves additional functions beyond mediating MLKL dimerisation, possibly by promoting transition to an active conformation, assembly of MLKL into higher order oligomers or complexes with other protein effectors.

These findings add an additional layer of regulation to our original model for MLKL activation. Building on our earlier findings,^25, 26, 28^ we hypothesise that HSP90 is required to effect the phosphorylation-induced conformational change in MLKL, to promote MLKL assembly into higher order complexes or to direct the activated MLKL to the membrane. Notably, in the current study, the stimulus-independent death mediated by the S345D MLKL mutant circumvented the requirement for upstream signals from RIPK1 and RIPK3, allowing us to directly implicate HSP90 as a regulator of MLKL activity. Each of these possibilities is supported by the literature, because HSP90 has been shown to promote conformational modifications,^63, 64, 65^ facilitating protein complex formation,^32, 66, 67^ and participating in intracellular trafficking^32, 50, 68^ of conventional kinases. Indeed, within the necroptosis pathway, HSP90 was recently reported to facilitate the interaction of RIPK3 with RIPK1 and RIPK3-mediated phosphorylation of MLKL.^42^

It is notable that the three proteins that directly contribute to necroptosis should be regulated by HSP90. RIPK1 was first identified in 2000 as an HSP90 client^39^ and, as has been shown in many reports, RIPK1 is rapidly degraded upon HSP90 inhibition.^36, 37, 38, 41^ RIPK3 was more recently shown to be an HSP90 client.^2, 31, 42, 43, 69^ In our hands, with three different inhibitors, loss of RIPK1 was more pronounced in U937 cells than in MDFs. Notably, RIPK1 levels are not always affected by HSP90 inhibition as shown in a recent study where it was proposed that activation of RIPK3 is regulated by HSP90.^42^ Ultimately, these studies illustrate that the effects of HSP90 inhibitors on RIPK1 and RIPK3 abundance, or necroptotic signalling, vary depending on the cell line examined. Here, we showed that HSP90 inhibitors rescued ~30% of mouse fibroblasts from death following exposure to a necroptotic stimulus. In contrast, mouse L929 cells exhibited ~60% protection from necroptotic death following treatment with HSP90 inhibitors,^43, 69^ whereas mouse macrophages were not protected by treatment with 17DMAG.^42^ Relatedly, we observed that HSP90 inhibitors were intrinsically more toxic to MDFs than U937 cells in our study, which impacted the relative protection from necroptotic death conferred by inhibiting HSP90.

Downstream of RIPK1 and RIPK3, we implicated MLKL as a direct target of HSP90, supported by the finding that killing by the auto-activating S345D mutant in *Ripk3*^−^^/−^*Mlkl*^−^^/^^−^ cells was strongly inhibited by three different HSP90 inhibitors. Although HSP90 inhibitors are effective killers of tumour cells and several are well advanced in clinical trials,^70^ the finding that they are also efficient cell death inhibitors is less surprising now that we have a greater understanding of their effects on levels and function of three effectors in the necroptosis pathway. Furthermore, it is tempting to speculate that necroptosis inhibition could have been involved in earlier cases where HSP90 inhibitors prevented cell death.^71^

Taken together, our data demonstrate an expanded role for HSP90 chaperone function in necroptotic signalling. Our observations that HSP90 inhibition prevented oligomerisation and/or membrane translocation of activated MLKL, but had no bearing on cell death induced by the isolated executioner domain encoded by the MLKL(1–180) construct, suggest that HSP90 may be involved in mediating or transmitting phosphorylation-induced conformational changes in the MLKL pseudokinase domain to facilitate higher order complex assembly and/or membrane translocation before necroptotic cell death. These results enhance our understanding of the cellular processes that underlie MLKL regulation and expand the repertoire of functions performed by HSP90 on pseudokinase clients.

## Materials and Methods

### Expression constructs

cDNAs encoding mouse MLKL(1–180), wild-type, Q343A or S345D mouse MLKL(1–464) were ligated into the doxycycline-inducible, puromycin-selectable vector, pF TRE3G PGK puro, as described previously.^25, 28^ A cDNA encoding wild-type mouse MLKL(1–464) lacking a stop codon was ligated in-frame upstream of the DNA gyraseB domain within a modified version of pF TRE3G PGK puro, as described elsewhere.^53^ All insert sequences were verified by sequencing (Micromon DNA Sequencing Facility, VIC, Australia). These constructs were used to generate lentiviral particles by co-transfection of HEK293T cells with vector DNA and pVSVg and pCMV ΔR8.2 helper plasmids as before.^25, 28^

### Antibodies, reagents and chemicals

The following primary antibodies were used for western blots of both mouse and human cell lines: anti-MLKL (produced in-house;^25^ available as MABC604, EMD Millipore, Billerica, MA, USA), anti-GAPDH (2118, Cell Signalling Technology, Danvers, MA, USA), anti-Actin (A-1987, Sigma-Aldrich, St Louis, MO, USA), anti-RIPK1 (610458, BD Biosciences, Franklin, NJ, USA), anti-Hsp90 (ADI-SPA-835, Enzo, Life Sciences, Farmingdale, NY, USA), anti-Cdc37 (D11A3, Cell Signalling Technology), and anti-VDAC (Sigma-Aldrich). Anti-mouse RIPK3 (PSC-2283-c100, Axxora, San Diego, CA, USA) and anti-human RIPK3 (ab56164, Abcam, Cambridge, UK) were used for their respective cell lines.

Recombinant hTNF-Fc, produced in-house, and the Smac mimetic, Compound A, were described previously.^71, 72^ QVD-OPh was obtained from R&D Systems (Minneapolis, MN, USA). Both NVP-BEP800 and 17-AAG were obtained from Selleckchem (Sydney, NSW, Australia), and AT13387 was produced by Active Biochem (Wanchai, Hong Kong).

### Phenotypic screen

Wild-type MDFs and *Mlkl*^−/−^ MDFs expressing Q343A mutant MLKL were assessed against a small-molecule library of 438 compounds consisting of 276 kinase inhibitors, 73 targeted agents and 89 epigenetic modulators. Assay-ready 384-well white tissue culture-treated plates (Greiner Bio-One, Kremsmünster, Austria) were prepared at Compounds Australia (Nathan, QLD, Australia) by transferring compound stocks or DMSO using an ECHO liquid handler (Labcyte, Sunnyvale, CA, USA). Cells were seeded into the plates at 2000 cells/well and pre-incubated with 1 *μ*M of inhibitor for 2 h before addition of either doxycycline (1 *μ*g/ml) or TNF (100 ng/ml), Smac mimetic (0.5 *μ*M) and QVD-OPh (5 *μ*M). After 24 h, cell viability was determined using CellTiter Glo (Promega, Madison, WI, USA) according to the manufacturer's instructions. Assays were performed in duplicate, and data were normalised to doxycycline-/TSQ-treated controls (0% viability) and DMSO-treated controls (100% viability) and expressed as percent rescue/viability. *Z*^′^ value (a measure of assay robustness) was >0.5 for all plates.

### Cell culture and cell death assays

U937 cells were maintained in human tonicity RPMI medium supplemented with 8–10% v/v fetal calf serum (FCS), as before.^51^ Experiments were carried out in 24-well plates, seeded at 1 × 10^5^ cells per well. For cell death assays, cells were pre-incubated with 17-AAG (500 nM), AT13387 (2 *μ*M), NVP-BEP800 (1 *μ*M) or DMSO for 1 h, then treated with TNF (100 ng/ml), Smac mimetic (500 nM) and QVD-OPh (10 *μ*M). After 24 h, cells were harvested, stained with propidium iodide (PI; 1 *μ*g/ml) and quantified using a FACSCalibur flow cytometer (BD Biosciences).

MDFs from the dermis of three *Mlkl*^−/−^ mice,^25^ three *Ripk3*^−/−^ mice (kindly provided by Dr Vishva Dixit^73^), three *Ripk3*^−/−^*Mlkl*^−/−^ mice^51^ and three congenic wild-type mice were isolated and immortalised to generate three biologically independent cell lines, as previously described.^25, 28^ MDFs were cultured in Dulbecco's modified Eagle's medium (DMEM) supplemented with 8–10% v/v FCS, with puromycin (2 *μ*g/ml) included for lines stably transduced with MLKL expression constructs. Cell death assays were carried out in 24-well plates, seeded at 5 × 10^4^ cells per well and allowed to attach overnight. MDFs were pre-incubated with 17-AAG (500 nM), AT13387 (2 *μ*M), NVP-BEP800 (1 *μ*M) or DMSO for 1 h, then treated with TNF, Smac mimetic and QVD-OPh, as described above for U937 cells. After 24 h, cells were harvested and quantified using flow cytometry as outlined above.

### Inhibitor assays and western blot

U937 cells, wild-type MDFs and *Ripk3*^−/−^ MDFs were treated with Hsp90 inhibitors as described above at indicated time points over a 24-h period. *Mlkl*^−/−^ and *Ripk3*^−/−^*Mlkl*^−/−^ cells with inducible MLKL expression constructs were also treated with doxycycline (50 ng/ml) 1 h after inhibitor treatment. Cells were harvested in RIPA lysis buffer in the presence of protease inhibitors, and total protein quantified using the bicinchoninic acid (BCA) microplate assay (ThermoScientific, Waltham, MA, USA) as per manufacturer's instructions. An equal amount of protein from each lysate was separated using SDS-PAGE and transferred onto PVDF membranes. These were incubated with primary antibodies as indicated above, HRP-conjugated secondary antibodies, and visualised with Immobilon Western Chemiluminescent HRP Substrate (EMD Millipore) using film or a ChemiDoc (Bio-Rad, Hercules, CA, USA).

### Fractionation and Blue Native PAGE

Cells were seeded into six-well plates at 2.5 × 10^5^ cells/well and allowed to settle overnight. *Ripk3*^−/−^*Mlkl*^−/−^ MDFs stably transduced with mutant MLKL S345D were pre-incubated for 1 h with 17-AAG, NVP-BEP800 or DMSO as described above, then induced with 50 ng/ml doxycycline for 7 h. In parallel, *Mlkl*^−/−^ MDFs reconstituted with full-length MLKL were induced with doxycycline, and after 3 h treated with TNF, Smac mimetic and QVD-OPh as described above, or left untreated for a further 5 h. Blue Native PAGE experiments were performed on U937 cells as described previously^51^ following pre-treatment of cells with DMSO, 17-AAG, AT13387 or NVP-BEP800 for 12 h and stimulation with TSQ for a further 12 h before lysis. Fractionation of cells into cytoplasmic and membrane fractions was performed as previously.^28, 51^ In brief, cells were permeabilised in buffer containing 0.025% digitonin (BIOSYNTH, Staad, Switzerland), 2 *μ*M N-ethyl maleimide, phosphatase and protease inhibitors. Permeabilised cells were centrifuged to separate crude membrane and cytosolic fractions, which were then respectively prepared in buffers to a final concentration of 1% w/v digitonin. Samples were separated on a 4–16% Bis-Tris Native PAGE gel, and transferred onto PVDF for western blot analyses.

### siRNA knockdown of Cdc37

*Mlkl*^−/−^ *Ripk3*^−/−^ MDFs stably infected with inducible MLKL S345D were seeded into 24-well plates in antibiotic-free DMEM at 2 × 10^4^ cells/well in the presence of either 1 *μ*l/ml DharmaFECT 1 transfection reagent (Dharmacon, Lafayette, CO, USA) and 40 nM of pooled siRNA, consisting of four murine Cdc37 targeted oligonucleotides (cat. L-060637, Dharmacon) or scrambled non-targeting siRNA (cat. D-001810-10, Dharmacon), transfection reagent alone, or left untreated. After 24 h, medium was changed to prevent transfection reagent toxicity. After a further 24 h, MLKL S345D expression was induced with 50 ng/ml doxycycline or cells were left untreated. For cell death assays, cells were harvested 24 h after doxycycline induction (72 h after original siRNA treatment) and percentage cell death assessed by propidium iodide uptake using flow cytometry. In parallel, duplicate wells were harvested using an SDS lysis buffer, and western blot was performed to verify knockdown of Cdc37 as described above.

### Sizing column fractionation of U937 cell lysates

In all, 30 × 10^6^ U937 cells were incubated for 18 h with DMSO or 17-AAG (500 nM) before lysis in 0.5 ml ice-cold modified DISC buffer (20 mM Tris-HCl pH 7.4, 150 mM NaCl, 10% glycerol, 0.5% Triton X-100) supplemented with Complete protease inhibitor cocktail (Roche, Basel, Switzerland). Lysate was clarified by centrifugation and the supernatant injected onto a Superose-6 10/300GL size-exclusion chromatography column pre-equilibrated with lysis buffer containing Complete protease inhibitor cocktail. Protein was eluted at a flow rate of 0.4 ml/min and 0.4 ml fractions collected. Samples were resolved by reducing SDS-PAGE on 4–12% Bis-Tris gels (Life Technologies, Carlsbad, CA, USA), before transfer onto PVDF for western blot analyses.

## Abbreviations

MLKL: mixed lineage kinase domain-like
4HB: four-helix bundle
RIPK: receptor interacting protein kinase
HSP90: heat-shock protein 90
Cdc37: cell division cycle 37
TNF: tumour necrosis factor
TNFR1: tumour necrosis factor receptor 1
TSQ: TNF (T), Smac mimetic Compound A (S), and QVD-OPh (Q)
PI: propidium iodide
MDF: murine dermal fibroblast
SEC: size-exclusion chromatography
DMEM: Dulbecco's modified Eagle's medium
FCS: fetal calf serum
