# Supplementary material for: HSP90 activity is required for MLKL oligomerisation and membrane translocation and the induction of necroptotic cell death
Source: Cell Death Dis. 2016 Jan 14;7(1):e2051–. doi: 10.1038/cddis.2015.386 (PMC4816171; doi:10.1038/cddis.2015.386)
Supplement: Supplementary Information [file cddis2015386x1.pdf]

Supplementary Figure Legends

Supplementary Figure 1. HSP90 inhibitors do not induce thermal shifts in MLKL. **(a-d)** Murine and human pseudokinase domain and full length MLKL protein were analysed using a thermal shift assay in the presence of 1 μM HSP90 inhibitors (AT13387, NVP-BEP800, or 17-AAG) or DMSO. Data are representative of at least two independent experiments.

Supplementary Figure 2. HSP90 inhibitor titrations. **(a-c)** Wild-type MDFs were treated with inhibitor followed by TSQ after 1 h, or inhibitor alone at the concentrations indicated. After 24 h, Propidium Iodide (PI) uptake was measured using flow cytometry. Two independent experiments were performed, each using three biologically independent cell lines (n = 6). Data are represented as the mean and ± standard error of the mean (SEM). **(d-f)** U937 cells were treated with indicated inhibitor followed by TSQ after 1 h, or inhibitor alone at the concentrations indicated. After 24 h, PI uptake was measured using flow cytometry. Three independent experiments were performed (n = 3). The curve fit was performed using non-linear regression.

Supplementary Figure 3. The HSP90 inhibitors, 17AAG and NVP-BEP800, prevent endogenous MLKL oligomerisation and membrane translocation despite the transitory nature of the MLKL:HSP90 interaction.

(**a**) Oligomerisation and membrane localisation of endogenous MLKL in TSQ-treated U937 cells was blocked by preincubation with 17-AAG (500 nM), NVP-BEP800 (1 μM), but not AT13387 (2 μM), or DMSO for 12 h. Cells were lysed and separated into cytoplasmic (C) and membrane (M) fractions before resolution of proteins by Blue-Native PAGE. Levels of MLKL were

analysed by Western blot. The high molecular weight MLKL-containing complex ("II") is observed upon membrane translocation, but only the low molecular species ("I") was observed in the cytoplasmic fraction. Success of fractionation was established using Western blot analysis of VDAC and GAPDH levels in the membrane and cytoplasmic fractions, respectively.

(**b**) S345D mMLKL bearing a C-terminal StrepII tag expressed in *Mlkl*$^{-/-}$ MDFs did not stably interact with HSP90 or Cdc37 in the presence or absence of TSQ treatment. Cells were lysed in MELB buffer supplemented with 0.05% digitonin and StrepII tagged species pulled down using StrepTactin resin. Bound proteins were eluted with 10 mM desthiobiotin (Elutions 1 and 2) or 100 mM glycine (pH 2; Elution 3) before beads were boiled in SDS loading buffer (Boiled beads). Proteins were resolved by SDS-PAGE before transfer to PVDF for examination by Western blot with the indicated antibodies.

Supplementary Methods

Recombinant protein expression and purification

Recombinant mouse (residues 179-464) and human (residues 190-471) MLKL pseudokinase domains and full length mouse MLKL (residues 1-464) bearing a conventional N-terminal His_6 tag as encoded by the pFastBac HTb vector were expressed and purified from Sf21 insect cells as described previously.[1,2] Briefly, these proteins were purified from Sf21 lysates by Ni^{2+}-affinity chromatography (Roche HisTag resin). The His_6 tag was then cleaved by incubation with TEV protease for 1 hour at 25°C, before extensive dialysis, further Ni^{2+}-chromatography to eliminate undigested protein and TEV protease followed by Superdex-200 gel filtration chromatography (GE Healthcare). Protein was eluted in 200 mM NaCl, 20 mM HEPES pH 7.5 for thermal

stability shift assays. Recombinant full length human MLKL (residues 2-471) bearing an N-terminal GST tag encoded by in-frame ligation into pFastBac GST was expressed and purified from Sf21 cells using standard methods.[2,3] Briefly, lysates were prepared in GST buffer (0.2M NaCl, 20mM HEPES pH 7.5, 5% v/v glycerol, 0.5mM TCEP) supplemented with Complete Protease Cocktail (Roche), clarified by centrifugation and filtration, and mixed with Glutathione-agarose resin (UBI Biosystems) at 4°C for 1 h. Beads were washed with GST buffer before incubation for 1 h at 25°C with TEV protease. Eluate, containing free full length human MLKL, was concentrated by centrifugal ultrafiltration and applied as above to a Superdex-200 gel filtration column. All proteins were concentrated by centrifugal ultrafiltration to >5mg/mL, aliquoted and snap frozen in liquid N_2, stored at -80°C until required.

Thermal shift assay for small molecule binding

Thermal shift assays were performed as described previously[1,2,4,5] using a Corbett Real Time PCR machine with proteins diluted in 150 mM NaCl, 20 mM Tris pH 8.0, 1 mM DTT to 2-5 μM and assayed in the presence of DMSO or 1 μM AT13387, 17-AAG or NVP-BEP800 in a total reaction volume of 25 μL. SYPRO Orange (Molecular Probes, CA) was used as a probe to monitor unfolding by measuring fluorescence at 530 nm. The temperature was raised in 1°C per minute steps from 25°C to 95°C and fluorescence readings at each degree step. Fluorescence of each sample was plotted as a function of increasing temperature and the melting temperature (T_m) corresponding to the midpoint for the protein unfolding transition was calculated by fitting the sigmoidal melt curve to the Boltzmann equation using GraphPad Prism. Positive changes in the unfolding transition temperature compared to the control curve (ΔT_m) were induced by presence

of a small molecule inhibitor would infer ligand binding to the protein. At least two independent assays were performed for each protein sample and representative data are shown for each.

Inhibitor Titrations

U937 cells were cultured in human tonicity RPMI medium supplemented with 8-10% v/v foetal calf serum (FCS), as previously.[6] Cells were seeded into 48 well plates at 5 x 10^4 cells per well for all experiments. To assess inhibitor toxicity, cells were treated with a two-fold dilution series of 17-AAG, AT13387, and NVP-BEP800, from 10 μM to 30 nM. In parallel, ability to inhibit necroptosis was assessed by addition of TNF (100 ng/mL), Smac-mimetic (500 nM) and QVD-OPh (10 μM) 1 h after inhibitor treatment. DMSO controls were included for all experiments. Cells were harvested after 24 h, stained with propidium iodide (PI; 1 μg/mL) and quantified using a BD FACSCalibur flow cytometer.

The dermis of three congenic wild-type mice were used for the isolation and immortalisation of murine dermal fibroblasts (MDFs) to generate three biologically independent cell lines, as previously described,[2,6] and maintained in Dulbecco's modified Eagle's medium (DMEM) supplemented with 8-10 % v/v FCS. Cells were seeded into 24 well plates at 5 x 10^4 cells per well and allowed to attach overnight for all experiments. Toxicity and inhibitor efficacy against TSQ mediated death were assessed as above for U937, with the exception that the serial dilution set covered the range of 10 μM to 125 nM. After 24 hours, cells were harvested and quantified using flow cytometry as outlined above.

Streptactin pulldown

Mlkl$^{-/-}$ *Ripk3*$^{-/-}$ MDFs stably infected with inducible MLKL S345D with a C-terminal StrepII-tag were left untreated, or induced with 10 ng/mL doxycycline in both the presence and absence of TSQ. After 4 h, cells were harvested with MELB lysis buffer (20 mM HEPES pH 7.5, 100 mM KCl, 2.5 mM MgCl2, 100 mM sucrose) supplemented with Complete Protease inhibitor cocktail (Roche) and 0.05% digitonin (BIOSYNTH, Staad, Switzerland). Clarified cell lysates were incubated with MagStrep "type2HC" beads (Iba Lifesciences) for 1 h at 4°C, then beads were washed five times with lysis buffer with additional Tween-20 (0.05%) or NaCl (300 mM total). Bound proteins were eluted twice with 10 mM desthiobiotin and a final elution performed with 100 mM glycine (pH 2.0). Beads were boiled in 2x SDS loading buffer to ensure complete protein removal. Collected protein was then analysed using Western blot analysis as described in the main text methods.

References

1 Murphy JM, Lucet IS, Hildebrand JM, Tanzer MC, Young SN, Sharma P *et al.* Insights into the evolution of divergent nucleotide-binding mechanisms among pseudokinases revealed by crystal structures of human and mouse MLKL. *Biochem J* 2014; **457**: 369–77.

2 Murphy JM, Czabotar PE, Hildebrand JM, Lucet IS, Zhang JG, Alvarez-Diaz S *et al.* The pseudokinase MLKL mediates necroptosis via a molecular switch mechanism. *Immunity* 2013; **39**: 443–453.

3 Cook WD, Moujalled DM, Ralph TJ, Lock P, Young SN, Murphy JM *et al.* RIPK1- and RIPK3-induced cell death mode is determined by target availability. *Cell Death Differ* 2014; **21**(10): 1600-1612.

4 Murphy JM, Zhang Q, Young SN, Reese ML, Bailey FP, Eyers P a *et al.* A robust methodology to subclassify pseudokinases based on their nucleotide-binding properties. *Biochem J* 2014; **457**: 323–34.

5 Hildebrand JM, Tanzer MC, Lucet IS, Young SN, Spall SK, Sharma P *et al.* Activation of the pseudokinase MLKL unleashes the four-helix bundle domain to induce membrane localization and necroptotic cell death. *Proc Natl Acad Sci* 2014; **111**: 15072–15077.

6 Tanzer MC, Tripaydonis A, Webb AI, Young SN, Varghese LN, Hall C *et al.* Necroptosis signalling is tuned by phosphorylation of MLKL residues outside the pseudokinase domain activation loop. *Biochem J* 2015; **471**(2): 255-265.

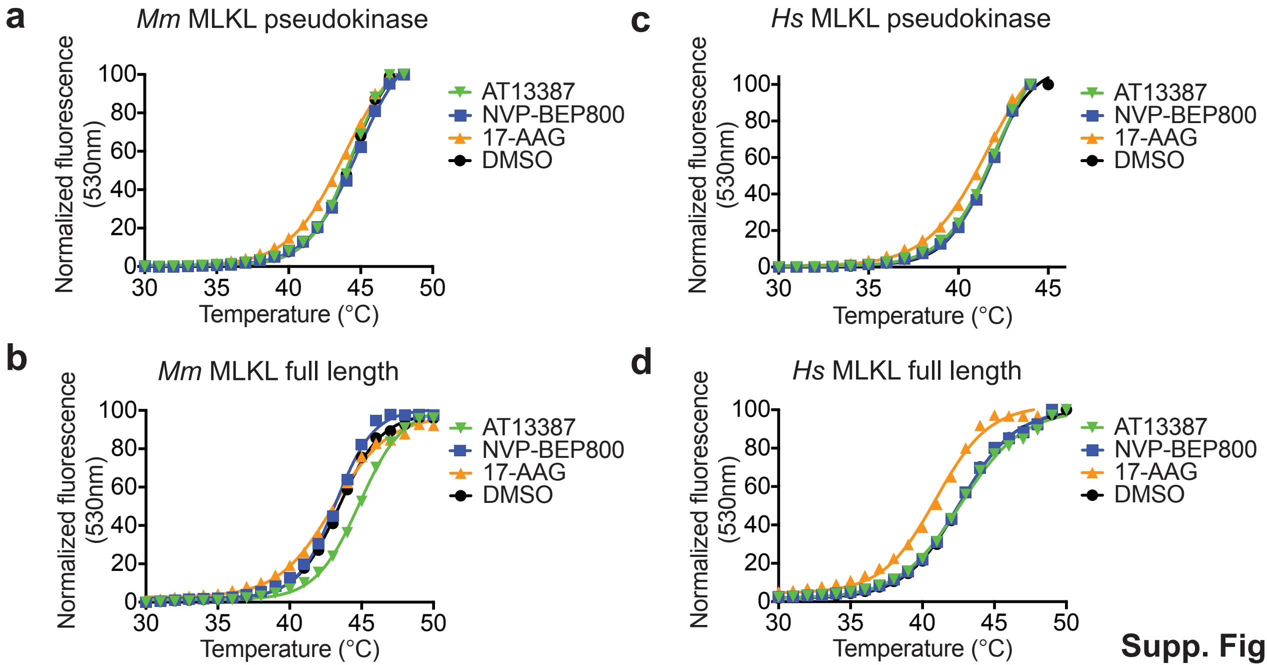

a
Mm MLKL pseudokinase
b
Mm MLKL full length
c
Hs MLKL pseudokinase
d
Hs MLKL full length
Normalized fluorescence (530nm)
Temperature (°C)
AT13387
NVP-BEP800
17-AAG
DMSO

Supp. Fig. 1

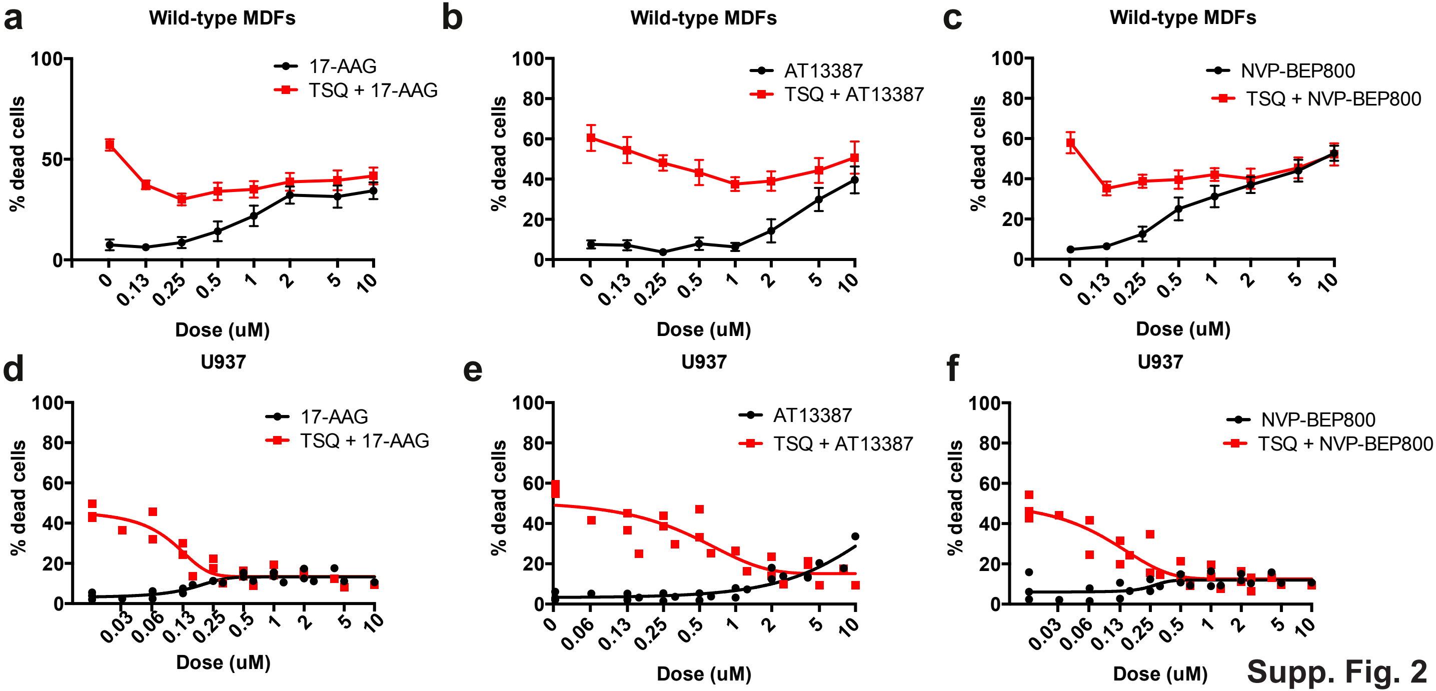

a
Wild-type MDFs
17-AAG
TSQ + 17-AAG
% dead cells
Dose (uM)
b
Wild-type MDFs
AT13387
TSQ + AT13387
% dead cells
Dose (uM)
c
Wild-type MDFs
NVP-BEP800
TSQ + NVP-BEP800
% dead cells
Dose (uM)
d
U937
17-AAG
TSQ + 17-AAG
% dead cells
Dose (uM)
e
U937
AT13387
TSQ + AT13387
% dead cells
Dose (uM)
f
U937
NVP-BEP800
TSQ + NVP-BEP800
% dead cells
Dose (uM)

Supp. Fig. 2

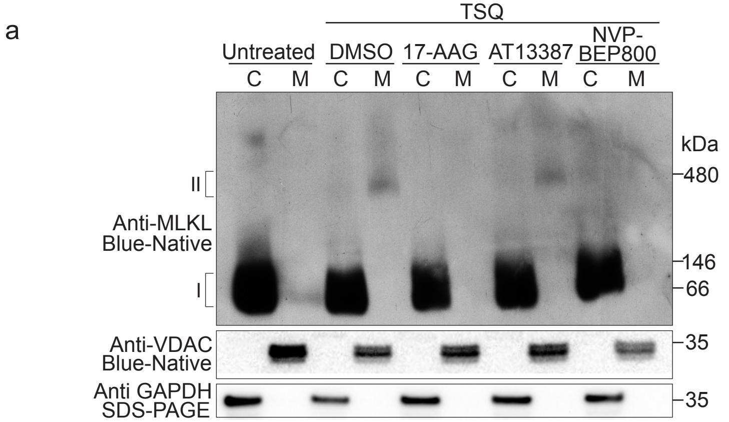

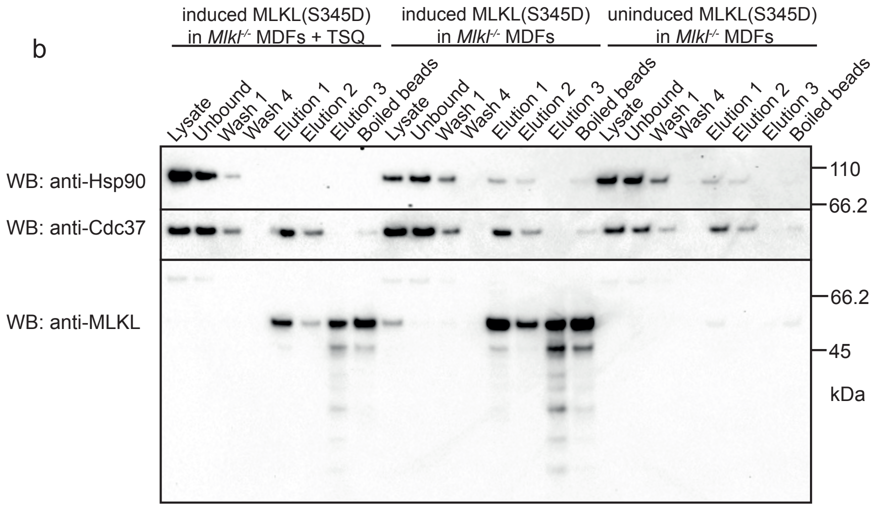

Supplementary Figure 3
